# Interactions of Surface Exopolysaccharides From *Bifidobacterium* and *Lactobacillus* Within the Intestinal Environment

**DOI:** 10.3389/fmicb.2018.02426

**Published:** 2018-10-11

**Authors:** Nuria Castro-Bravo, Jerry M. Wells, Abelardo Margolles, Patricia Ruas-Madiedo

**Affiliations:** ^1^Microhealth Group, Department of Microbiology and Biochemistry of Dairy Products, Instituto de Productos Lácteos de Asturias – Consejo Superior de Investigaciones Científicas (IPLA-CSIC), Villaviciosa, Spain; ^2^Host-Microbe Interactomics Group, Animal Science Department, Wageningen University and Research (WUR), Wageningen, Netherlands

**Keywords:** exopolysaccharide, intestinal-receptors, innate-immunity, adhesion, microbiota

## Abstract

Exopolysaccharides (EPS) are surface carbohydrate polymers present in most bacteria acting as a protective surface layer but also interacting with the surrounding environment. This review discusses the roles of EPS synthesized by strains of *Lactobacillus* and *Bifidobacterium*, many of them with probiotic characteristics, in the intestinal environment. Current knowledge on genetics and biosynthesis pathways of EPS in lactic acid bacteria and bifidobacteria, as well as the development of genetic tools, has created possibilities to elucidate the interplay between EPS and host intestinal mucosa. These include the microbiota that inhabits this ecological niche and the host cells. Several carbohydrate recognition receptors located in the intestinal epithelium could be involved in the interaction with bacterial EPS and modulation of immune response; however, little is known about the receptors recognizing EPS from lactobacilli or bifidobacteria and the triggered response. On the contrary, it has been clearly demonstrated that EPS play a relevant role in the persistence of the producing bacteria in the intestinal tract. Indeed, some authors postulate that some of the beneficial actions of EPS-producing probiotics could be related to the formation of a biofilm layer protecting the host against injury, for example by pathogens or their toxins. Nevertheless, the *in vivo* formation of biofilms by probiotics has not been proved to date. Finally, EPS produced by probiotic strains are also able to interact with the intestinal microbiota that populates the gut. In fact, some of these polymers can be used as carbohydrate fermentable source by some gut commensals thus being putatively involved in the release of bacterial metabolites that exert positive benefits for the host. In spite of the increasing knowledge about the role that these surface molecules play in the interaction of probiotic bacteria with the gut mucosal actors, both intestinal receptors and microbiota, the challenging issue is to demonstrate the functionality of EPS *in vivo*, which will open an avenue of opportunities for the application of EPS-producing probiotics to improve health.

## Bacterial polysaccharides

Microorganisms synthesize a wide variety of carbohydrates such as storage polymers (glycogens) located into the cytoplasm or structural polymers (glycans) which form part of the microbial envelope. Glycan polymers can be tightly linked to the cell surface forming a capsular polysaccharide, (CPS), loosely attached to the extracellular surface, or secreted to the environment as exopolysaccharides (EPS). In bacteria these polymers have received special attention because they play a relevant role in the interaction between pathogens and the host, being in some cases virulent factors responsible of pathogenesis (Poole et al., 2018). However, polymers synthesized by specific strains of commensal gut bacteria, such the surface polysaccharide A (PSA) produced by *Bacteroides fragilis* (Round et al., 2011), and the EPS of *Faecalibacterium prausnitzii* strain HTF-F (Rossi et al., 2015) can modulate intestinal immunity. In addition to their biological functions, bacterial EPS are being extensively studied because industrial, food, cosmetics or medical uses to highlight some of their applications (Rehm, 2010; Freitas et al., 2011; Moscovici, 2015; Gupta and Diwan, 2017). Thus, the knowledge of biosynthesis pathways and strategies to design tailor-made polymers (Schmid et al., 2015) as well as the optimization of purification processes (Donot et al., 2012) are needed to increase the range of applications.

**Figure 1:**
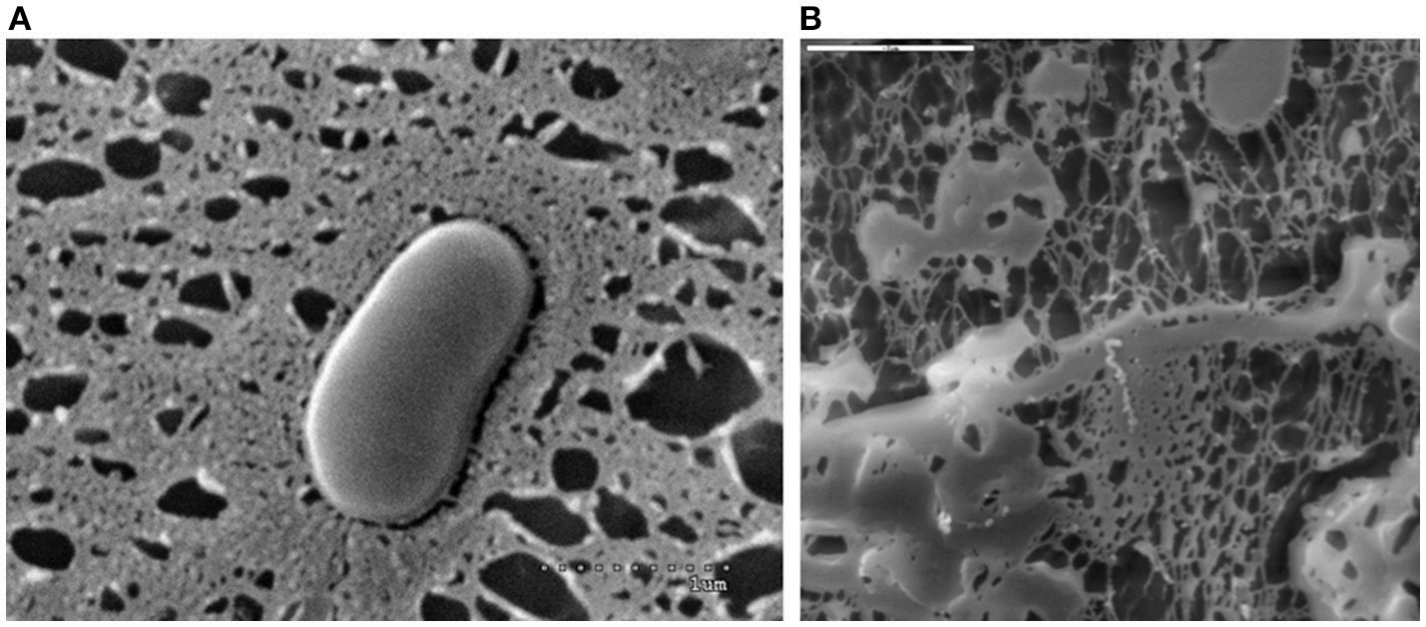
EPS-producing *Bifidobacterium animalis* subsp. *lactis* S89L visualized with a cryo-scanning electron microscope (**A** dotted line 1 μm) and EPS material released from the bacterium (**B** solid line 5 μm).

Lactic acid bacteria (LAB) are a particular group of Gram-positives that are involved in many natural food fermentations but there are also widely used in food manufacture industry. In addition, a few species of this group mainly those belonging to genus *Lactobacillus* (phylum *Firmicutes*), together with some from genus *Bifidobacterium* (phylum *Actinobacteria*) are used in the formulation of foods that promote health, which are known as functional foods (Linares et al., 2017). Some strains from these genera are able to synthesize and release EPS in the environment. Such strains could be easily detected after a direct macroscopic observation of the colonies growing in the surface of culture medium; they appear as regular, smooth, and glistening colonies some of them having a “ropy” phenotype denoted for the formation of a long filament when the colony is touched. Besides, the EPS surrounding the bacterium, or the polymer totally secreted to the environment, can be visualized using different microscopic techniques (Figure 1).

EPS-producing LAB have a long tradition of use in dairy fermentations because the polymers help to improve the texture, mouthfeel, and viscosity of fermented milks, being also effective fat-replacers, avoiding syneresis, or whey separation (Mende et al., 2016; Zannini et al., 2016). Positive attributes have been reported for other non-dairy foods (Ryan et al., 2015; Torino et al., 2015), although in some alcoholic-fermented beverages the presence of EPS could lead to sensorial alterations (Caggianiello et al., 2016). Thus, research on EPS-producing LAB, that are being continuously isolated and characterized from natural sources, are of ongoing interest to the food industry (Zeidan et al., 2017).

In addition to these technological applications, EPS are claimed to have pivotal role in the interaction of lactobacilli and bifidobacteria with the host. These polymers will act as one of the effector molecules involved in the dialog stablished at the gut level among probiotics, the intestinal mucosa and the microbiota inhabiting this niche. Therefore, the health properties of EPS-producing probiotic bacteria are being also extensively studied (Hidalgo-Cantabrana et al., 2014; Ryan et al., 2015; Lebeer et al., 2018). This is the topic that will be updated and discussed in the next sections.

## Genetics and synthesis of EPS in *Bifidobacterium* and *Lactobacillus*

**Figure 2:**
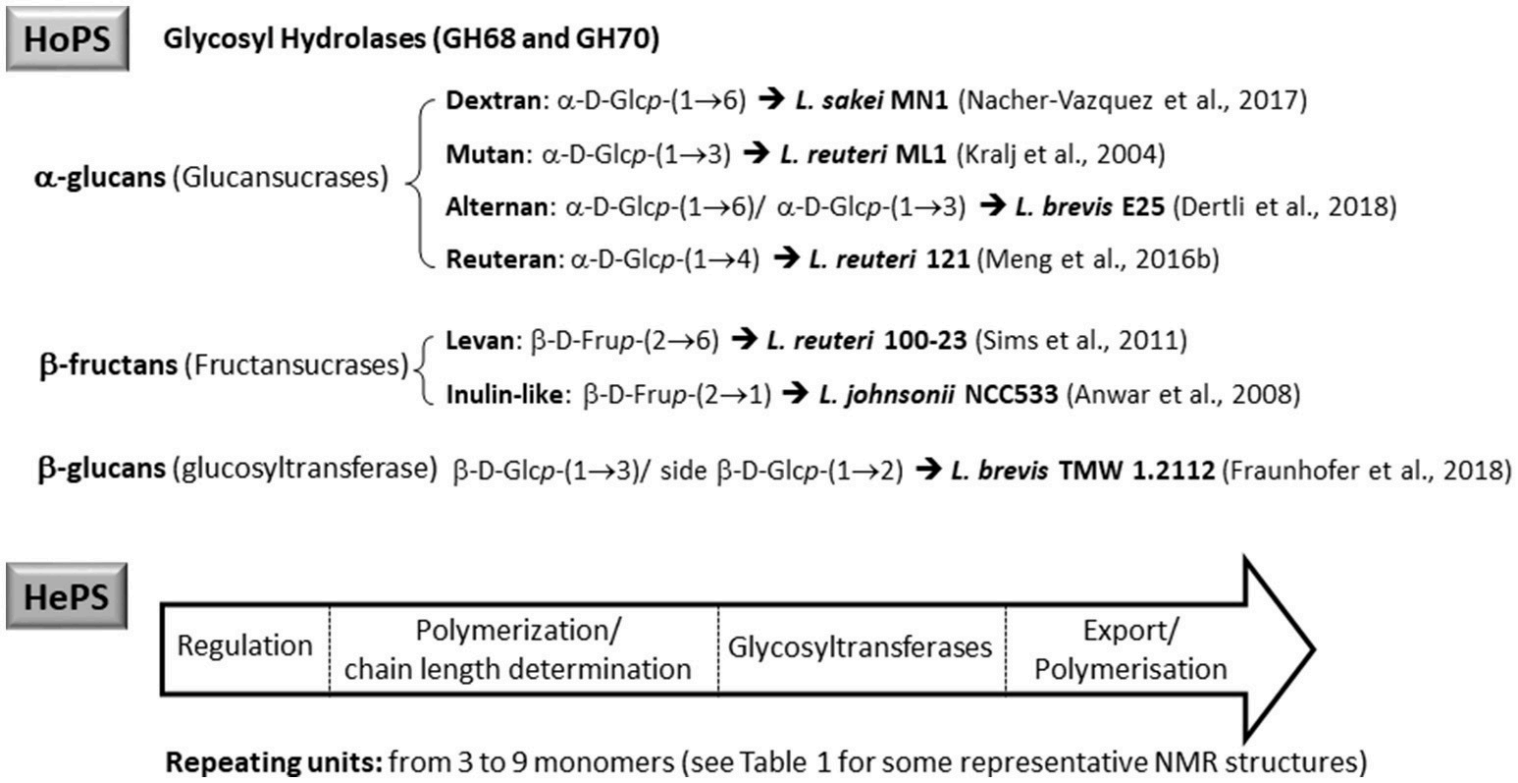
Main types of homopolysaccharides (HoPS) and schematic representation of the enzymes involved in the synthesis of heteropolysaccharides (HePS).

According to the chemical composition and the way of synthesis two types of EPS, homopolysaccharides (HoPS) and heteropolysaccharides (HePS), are synthesized by LAB or bifidobacteria (Hidalgo-Cantabrana et al., 2014; Torino et al., 2015; Caggianiello et al., 2016; Zannini et al., 2016). In short, HoPS are composed of glucose or fructose and, depending on the position of the carbon involved in the linkage, they could be α-glucans, β-glucans, or β-fructans (Figure 2) (Kralj et al., 2004; Anwar et al., 2008; Sims et al., 2011; Meng et al., 2016b; Nácher-Vázqueza et al., 2017; Dertli et al., 2018). Sucrose is the donor substrate for glycosyl hydrolase (GH) enzymes, named glucansucrases (family GH70), and fructansucrases (GH68) that catalyze the synthesis of α-glucans or β-fructans, respectively, and are located extracellularly (van Hijum et al., 2006; Meng et al., 2016a; Gangoiti et al., 2018; Yan et al., 2018). However, β-glucans are synthesized by means of glucosyltransferases from activated UDP-glucose acting as the donor instead of sucrose (Werning et al., 2006; Dols-Lafargue et al., 2008; Fraunhofer et al., 2018; Puertas et al., 2018). Among LAB found in foods, homopolymer-producers have been described for the genera *Streptococcus, Leuconostoc, Lactococcus, Pediococcus, Oenococcus*, and *Weisella* (Zannini et al., 2016); in the case of *Lactobacillus* (Figure 2) a number of species are able to produce α-glucans (dextran, mutan, and reuteran), β-glucans, and β-fructans (levan and inulin-like; Oleksy and Klewicka, 2018). In contrast, there are no published studies on the synthesis and release to the exocellular environment of HoPS in the genus *Bifidobacterium*.

In both, lactobacilli and bifidobacteria, HePS comprise repeating units of monosaccharides, mainly D-glucose, D-galactose, and L-rhamnose. Occasionally, N-acetylated monosaccharides, such as N-acetyl-glucosamine (GluNAc) and N-acetyl-galactosamine (GalNAc), fucose, glucuronic acid, glycerol, or mannose can also be present (Badel et al., 2011; Hidalgo-Cantabrana et al., 2014). The chemical characterization of these heteropolymers involves the use of different techniques to determine the monomer composition, the linkage site and branching pattern, among other features. The final structure of the repeating units is elucidated by means of nuclear magnetic resonance (NMR; Hidalgo-Cantabrana et al., 2012). In fact, more than fifty different NMR-EPS repeating unit structures have been described to date for lactobacilli and bifidobacteria (Table 1; update from Ruas-Madiedo et al., 2009, 2012; Castro-Bravo et al., 2018; Jiang and Yang, 2018; Oleksy and Klewicka, 2018). Unique structures, from three to nine monomers, were reported and they are not linked at the genus or species level, but each repeating unit is dependent on the producing strain.

**Table 1:** Update of novel repeating unit structures of EPS synthesized by *Bifidobacterium* and *Lactobacillus* determined by NMR techniques (update from Ruas-Madiedo et al., 2009, 2012; Castro-Bravo et al., 2018; Jiang and Yang, 2018; Oleksy and Klewicka, 2018).

**Species (culture media)**	**Strain**	**NMR-structure**	**References**
*B. longum* (MRS + 0.25% L-cystein)	W11	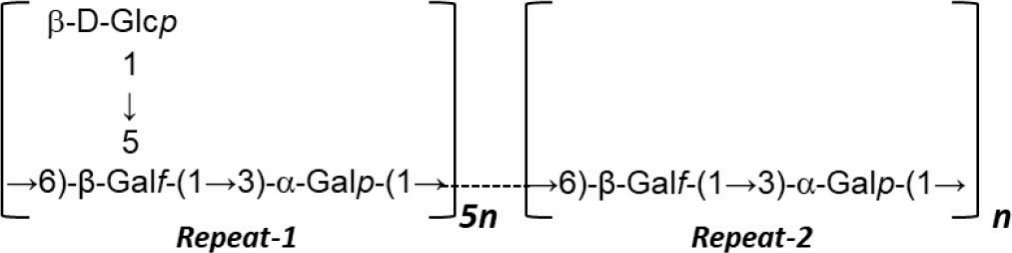	Inturri et al., 2017
*L. acidophillus* (MRS)	20079	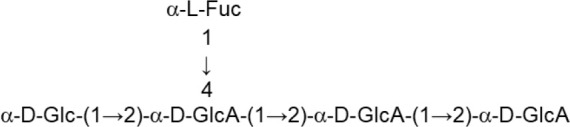	El-Deeb et al., 2018
*L. casei* (MRS)	BL23 variant 1	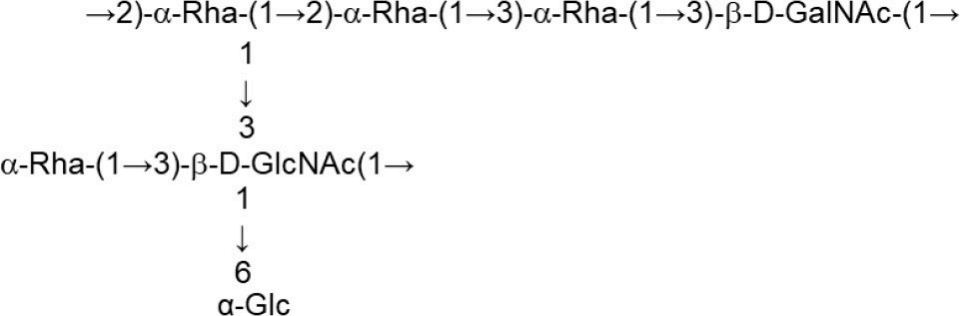	Vinogradov et al., 2016
*L. crispatus* (Semi-defined MRS supplemented with glucose or sucrose)	L1	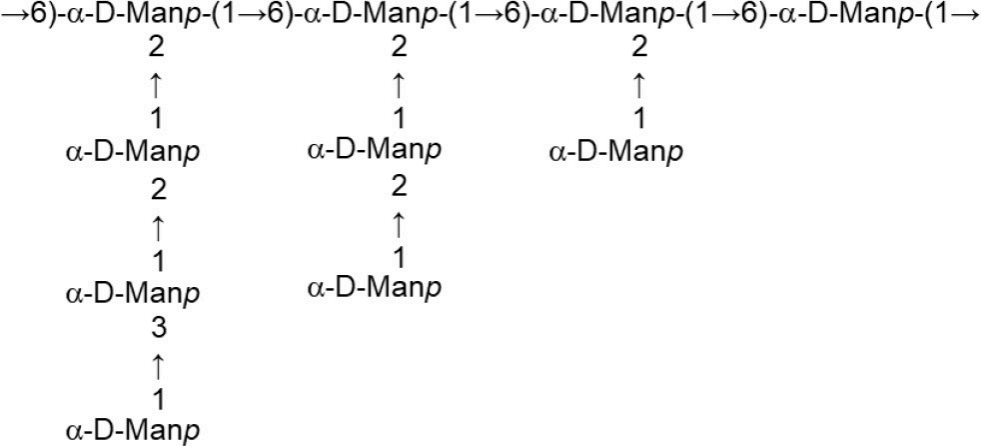	Donnarumma et al., 2014
*L. delbrueckii* subsp. *bulgaricus* (Skimmed milk)	LBB.B26	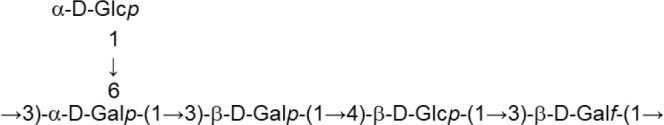	Sánchez-Medina et al., 2007
*L. delbrueckii* subsp. *bulgaricus* (Skimmed milk)	OLL1073R-1	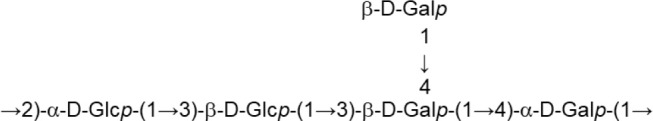	Van Calsteren et al., 2015
*L. fermentum* (MRS or Chemically defined medium supplemented with glucose)	TDS030603	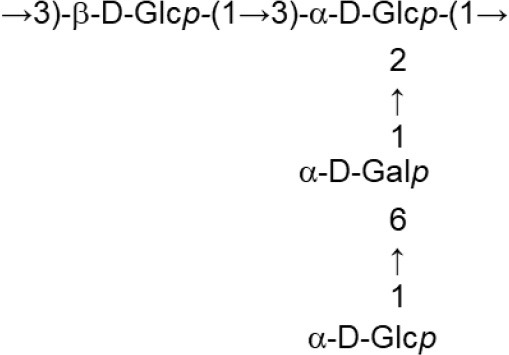	Gerwig et al., 2013
*L. helveticus* (Whey medium)	MB2-1 (fraction LHEPS-1)	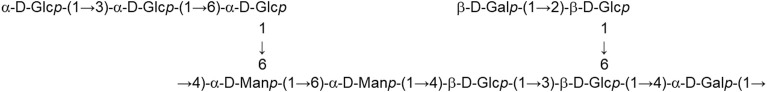	Li et al., 2015a
*L. helveticus* (Whey medium)	MB2-1 (fraction LHEPS-2)	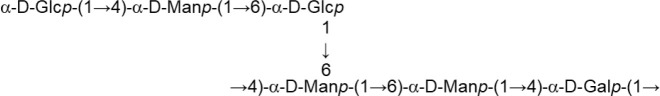	Li et al., 2014
*L. helveticus* (Whey medium)	MB2-1 (cell-wall)	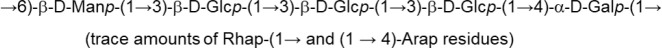	Li et al., 2015b
*L. johnsonii* (MRS)	142	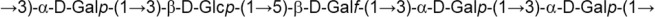	Górska et al., 2010
*L. johnsonii* (MRS)	151	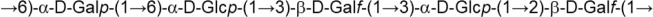	Górska-Fraczek et al., 2013
*L. johnsonii* (MRS supplemented with glucose)	FI9785 (fraction EPS1)	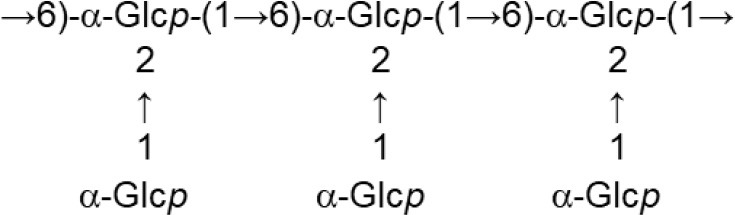	Dertli et al., 2013
	FI9785 (fraction EPS2)	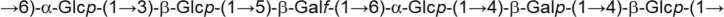	
*L. paracasei* (Chemically defined medium supplemented with glucose)	DG	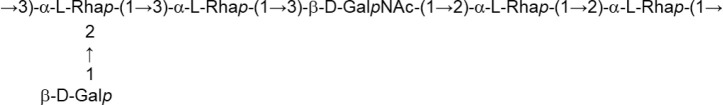	Balzaretti et al., 2017
*L. pentosus* (Modified semi-defined medium supplemented with glucose)	LPS26 (fraction EPS A)	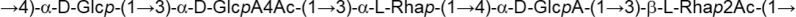	Rodríguez-Carvajal et al., 2008
*L plantarum* (MRS)	MTCC9510	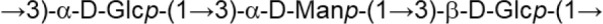	Ismail and Nampoothiri, 2010
*L. rhamnosus* (MRS)	KL37B	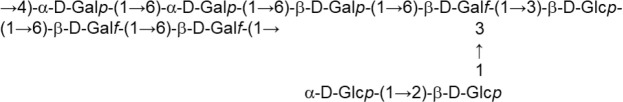	Górska-Fraczek et al., 2011

*Glc, glucose; Gal, galactose; Rha, rhamnose; Fuc, fucose; Man, mannose; GalNAc, N-acetyl-galactosamine; GlcNAc, N-acetyl-glucosammine; p, pyranose ring; f, furanose ring*.

The HePS biosynthesis is more complex than that of HoPS as it involves the activity of several genes, organized in *eps-*clusters, which have similar predicted functions in bifidobacteria and lactobacilli (Hidalgo-Cantabrana et al., 2014). The *eps-*clusters harbor genes coding for glycosyltranserases (GTF), involved in the biosynthesis of the repeating units, proteins related to polymerization and export of these units, as well as other genes with unknown functions including mobile elements. In addition, genes involved in the regulation of HePS synthesis are detected in lactobacilli but not in bifidobacterial *eps* clusters (Hidalgo-Cantabrana et al., 2014; Ferrario et al., 2016; Jiang and Yang, 2018). Another remarkable difference between both genera is that, in general, *eps* clusters in *Lactobacillus* have a conserved functional operon-like structure with genes oriented in one direction, whereas this is not found in *Bifidobacterium*. As an illustrative example, in the genome analysis of forty-three *Lactobacillus plantarum* strains four different *eps* gene clusters were distinguished. The strains that shared a given EPS type also shared the same structural organization of this *eps* cluster (Jiang and Yang, 2018). In contrast, no common structural organization was identified in the *eps-*clusters of five *Bifidobacterium longum* subsp. *longum* strains (Hidalgo-Cantabrana et al., 2014). Therefore, a conserved “consensus” *eps*-cluster for *Lactobacillus*, and other LAB (Figure 2), was proposed starting (from 5' to 3') with genes for transcriptional regulation, chain-length determination, a core of GTF and ending with genes for polymerisation/ export, all flanked by transposases (and derivatives) or insertion sequences (Hidalgo-Cantabrana et al., 2014). It is worth noting that the function of these genes has been mostly proposed by means of *in silico* analyses after homology comparison with the information held in genetic databases (Hidalgo-Cantabrana et al., 2014; Ferrario et al., 2016; Jiang and Yang, 2018). For only a few genes has the function been demonstrated by means of functional assays; examples of functionally characterized *eps* genes include the GTF (Lamothe et al., 2002) also the priming-GTF (Lebeer et al., 2009; Fanning et al., 2012; Tahoun et al., 2017), chain-length determining genes (Bouazzaoui and LaPointe, 2006; Hidalgo-Cantabrana et al., 2015), and transcriptional regulators of the *eps* operons (Dertli et al., 2016).

The pathway proposed for the synthesis of HePS in *Lactobacillus* and *Bifidobacterium* (Hidalgo-Cantabrana et al., 2014; Castro-Bravo et al., 2018; Oleksy and Klewicka, 2018) has been inferred from the predicted *eps* gene functions, which have been mostly studied in Gram-negative (O-antigen synthesis of lipopolysaccharides, Reeves and Cunnen, 2009) or in both Gram-positive and negative pathogens (capsular polysaccharides, Reid and Szymanski, 2009). In short, it seems that the first step in the HePS synthesis involves the intracellular build-up of individual repeating units that is initiated by the priming-GTF. This enzyme links the first monosaccharide to a lipidic carrier (undecaprenyl phosphate C55-P) located in the cytoplasmic membrane. Later, more sugar moieties are added by the sequential activity of different GTF that catalyze the glycosydic linkage between monosaccharides coming from activated nucleotide-sugar precursors. These precursors derived from the conversion of glucose, galactose, or lactose through intermediates of central metabolic pathways, such as glucose-1-phosphate and fructose-6-phosphate (De Vuyst et al., 2001). Then, these units are secreted across the membrane, and extracellularly polymerized, which could be achieved by means of two putative secretion-polymerization systems: i) ABC transporters and ii) the “flippase”-polymerase complex (also named Wzx/Wzy-dependent pathway, according to the nomenclature described for *E. coli*; Schmid, 2018). The *in silico* analysis of bifidobacterial *eps*-clusters revealed that both systems could be present whereas, as far as we could know, only the flippase-like export was described in lactobacilli (Hidalgo-Cantabrana et al., 2014). In the Wzx/Wzy-dependent pathway, once the undecaprenyl-phosphate-linked repeating units are assembled, they are “flipped” across the membrane by means of the Wzx (flippase) and the Wzy (polymerase) would extracellularly transfer the repeating unit to a growing chain, the final length of the polymer being determined by means of the Wzz (a tyrosine kinase). Indeed, it was demonstrated that mutations in genes coding for proteins (homologous to Wzz) harbored in *eps*-clusters from lactobacilli (Horn et al., 2013) and bifidobacteria (Castro-Bravo et al., 2017) are responsible for the modification of the molecular weight of the HePS synthesized by these bacteria. Finally, genes with homology to ABC-transporters have been found in *Bifidobacterium eps*-clusters (Ferrario et al., 2016); although it is unknown whether they are involved in the export of the HePS subunits, it can be hypothesized that this system could participate in the formation of heteropolymers in those bifidobacterial taxa for which the priming-GTF was not detected. Nonetheless, the biosynthetic route of HePS formation in *Lactobacillus* and *Bifidobacterium* is still poorly understood. In the case of pathogens synthesizing CPS is not clear which is the acceptor molecule for the first sugar, but it seems that is non-dependent on the lipidic carrier; then more sugar nucleotides, from nucleotide-activated precursors, are added to the non-reducing end of the chain forming the polymer which is transported through the membrane to the periplasmic side by means of the ABC-transporter in Gram-negative bacteria (Cuthbertson et al., 2009).

## EPS interactions with the intestinal mucosa

### Recognition of bacterial glycans by human C-type lectins

The innate recognition of microorganisms is mediated by several classes of pattern-recognition receptors, including Toll-like receptors (TLRs), nucleotide oligomerization domain-like receptors (NODs), RIG-1-like receptors (RLRs) and lectin receptors recognizing glycan-containing molecular structures. There are three classes of innate lectin receptors; the galectins which bind to beta-galactosidase sugars, the siglecs which bind to sialic acid containing glycans, and the C-type lectin receptors (CLRs) which bind to various glycans. The CLRs belong to the superfamily of C-type lectins comprising more than 1,000 proteins with a common protein fold known as the carbohydrate recognition domain (CRD) and have diverse functions in homing and trafficking of immune cells, T cell activation, innate recognition of microbes and regulation of immune responses.

The secreted soluble CLRs involved in innate immunity include SP-A, SP-D, mannose binding lectins (MBLs) also termed mannose-binding protein (MBP), that can bind to microbes and promote direct or indirect killing, phagocytic uptake, or activation of the lectin pathway of complement activation (Garred et al., 2016; Hansen et al., 2016). Several transmembrane signaling CLRs are expressed on the cell surface of the myeloid lineage of immune cells including dendritic cells, monocytes and macrophages where they can trigger signaling pathways involved in antimicrobial immunity (Mayer et al., 2017; Brown et al., 2018). Results of quantitative real-time PCR expression of CLRs in organs of mice and humans has been described (Lech et al., 2012). The organ- and species-specific expression of C-type lectin receptors was different between mice and humans raising the need for caution when translating results from mice to humans.

**Figure 3:**
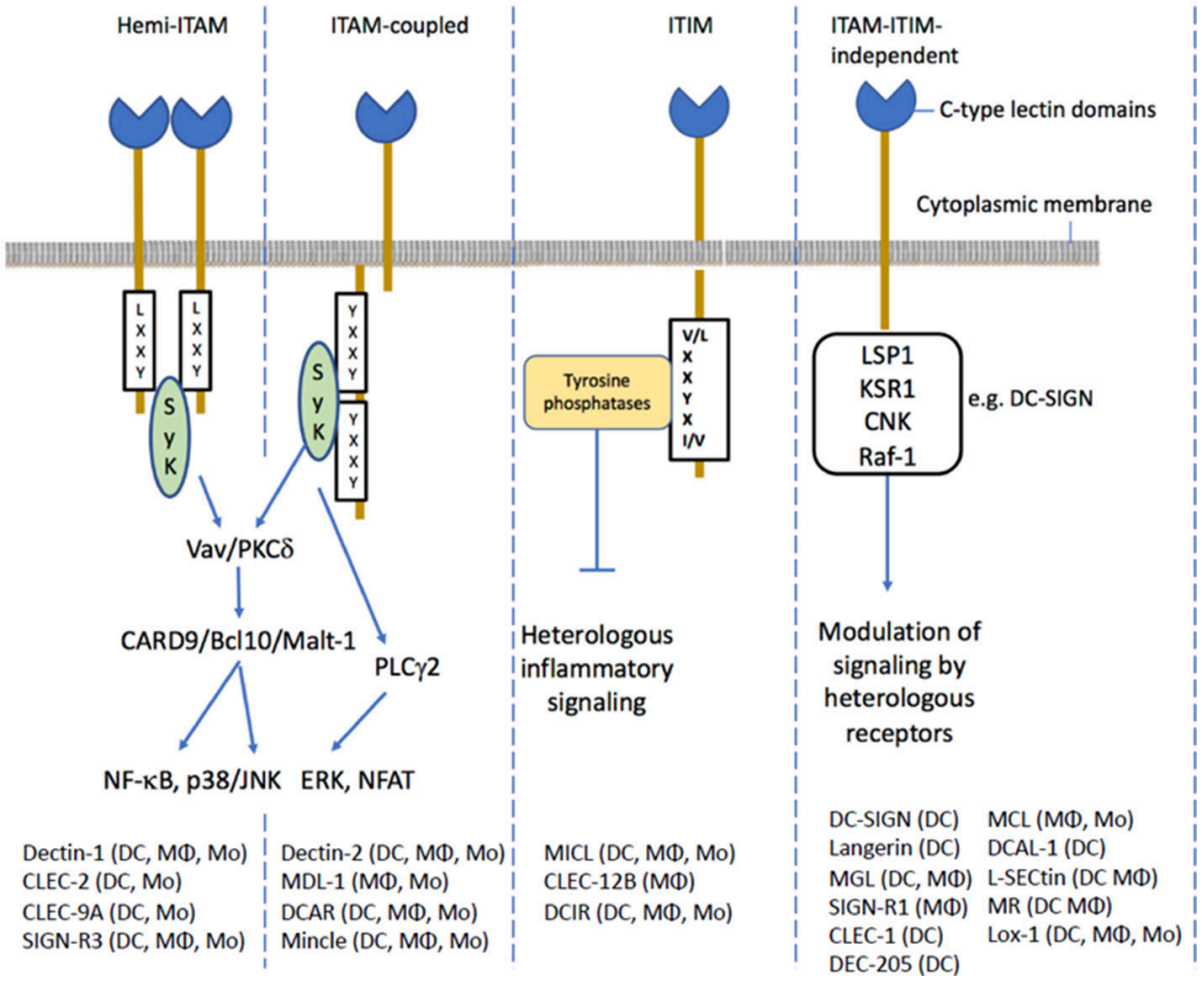
The membrane signaling CLRs can be assigned to different categories based on the modes of intracellular signaling. The ITAM containing CLRs either have tyrosine activating motifs in their cytoplasmic domains or interact with an ITAM-containing adapter protein. The hemi-ITAM CLRs require the interaction of two CLRs for signaling. The ITAM motif recruits Syk activating the CARD9/Bcl10/Malt-1 module to promote NF-κB signaling and expression of inflammatory genes. Dectin-1, an ITAM containing CLR has been shown activate ERK and NFATR through PLCγ2. The ITIM-containing CLRs recruit tyrosine phosphatases such as SHP-1 or SHP-2 which negative regulate heterologous inflammatory signaling pathways. Several CLRs do not possess ITAM or ITIM motifs in their cytoplasmic tails. For example, ligand binding to DC-SIGN results in formation of a signaling complex containing LSP1, KSR1, and CNK and the kinase Raf-1 which modulates inflammatory signaling through its acetylation of the p65 subunit of NF-κB.

One of the best characterized signaling CLRs involved in microbial recognition and immunity is Dectin-1 which is important for innate immunity to fungal pathogens (reviewed in Plato et al., 2013). Ligand-mediated dimerization of Dectin-1 enables the hemi-immune-receptor tyrosine-based activation motif (ITAM) to bind an intracellular tyrosine kinase (known as spleen tyrosine kinase; SYK) and formation of a signaling complex that activates NF-kB pathway leading to various cellular responses. The transmembrane C-type lectin dendritic cell (DC) immunoreceptor (DCIR), is expressed on dendritic cells and is an example of a signaling CLR receptor containing a tyrosine-based inhibitory motif (ITIM). Ligand binding to ITIM containing receptors recruits the tyrosine phosphatases (SHP1 and SHP2) which modulate immune signaling through other pattern-recognition receptors (Geijtenbeek and Gringhuis, 2009). For example, triggering human DCIR signaling with antibody inhibits TLR-mediated production of inflammatory cytokines (Meyer-Wentrup et al., 2008, 2009; Zhao et al., 2015). Many CLRs signal independently of ITAM and ITIM motifs, for example DC-SIGN can inhibit leukocyte activation via specific Raf-1-dependent signaling pathway which also modulates TLR-induced NF-κβ activation (Geijtenbeek et al., 2000; Gringhuis and Geijtenbeek, 2010). The ligand specificity and binding mode has only been characterized for a few CLRs involved in innate immunity, of which the best studied are DC-SIGN, Dectin-1, Dectin-2, or Mincle (Figure 3). Dectin-1 binds to β-1,3 glucans found in fungal cell walls and plays an important role in antifungal immunity. Dectin-2 is expressed on macrophages and dendritic cells and is another ITAM-bearing CLR. Dectin-2 recognizes α-mannan in fungal cell walls, mannose-capped lipoarabinomannan of *Mycobacterium tuberculosis* and mannosylated O-antigens of Gram-negative bacterial lipopolysaccharides. Another ITAM-containing CLR is Mincle which is expressed on monocytes, macrophages, dendritic cells and neutrophils. Mincle appears to bind to a broad range of bacteria pathogens including *Mycobacterium* spp., *Klebsiella pneumoniae, Streptococcus pneumonia*, and *Helicobacter pylori*. DC-SIGN is mainly expressed by myeloid and monocyte-derived DCs and binds preferentially to high mannose structures and fucose-terminated glycan structures. Several pathogens have been reported to bind DC-SIGN including human immune-deficiency virus, *M. tuberculosis* and *H. pylori*. Signaling through DC-SIGN on DCs and macrophages has an anti-inflammatory role enhancing IL-10 production, the generation of Treg cells and down-regulating pro-inflammatory responses (Geijtenbeek et al., 2003; Bergman et al., 2004).

There are few studies about glycan-mediated innate responses in intestinal epithelial cells with the exception of Dectin-1. This CLR is expressed in primary human enterocytes and responds to fungal β-glucans by inducing expression of chemokines (Cohen-Kedar et al., 2014). Furthermore, β-glucans in food have been shown to modify colonic microbiota by inducing antimicrobial protein, calprotectin, in a Dectin-1-induced-IL-17F-dependent manner (Kamiya et al., 2018).

Whether or not bacterial EPS activate or inhibit innate immunity via the transmembrane CLRs is not yet understood but recent progress in developing CLR-Fc fusion proteins may be useful to screen for EPS/CLR interactions in the future (Mayer et al., 2018).

### EPS and the immune response

A growing number of publications report *in vivo* and *in vitro* immune-stimulatory or immune-modulating effects of EPS from strains of *Lactobacillus* and *Bifidobacterium* which are too numerous to describe individually (Laiño et al., 2016). One example of an *in vivo* study in mice orally gavaged with isogenic EPS-producing and non-EPS-producing strains of *Bifidobacterium breve* for three consecutive days resulted in differences in mucosal immune cell populations. Additionally, fecal IgA and serum IgG were elevated in mice given the non-EPS-producing strains which correlated with other data on the role of EPS in colonization and, or the shielding effect of EPS on phagocytosis, immune activation and antigen presentation (Fanning et al., 2012).

Many studies *in vitro* have compared cytokine responses to EPS and non-EPS producing strains as well as “purified” (extracted) EPS (Hidalgo-Cantabrana et al., 2012). In the last case, the degree of purity of the polymers was uncertain since, in most cases, standard procedures were used for EPS isolation which could favor the co-precipitation of other bacterial components with immunogenic properties. Then, not all studies on immune responses to “purified” EPS test for contamination with other molecular associated microbial patterns (MAMPs) such as lipoteichoic acid, or lipoprotein anchors which could also activate immune cells via TLR2/1 and TLR2/6 receptors (Wells, 2011). One recent study, measured cytokine responses of bone-marrow derived DCs (BM-DCs) stimulated with EPS producing commensal (lactobacilli) bacteria from mice or the purified EPS (Górska et al., 2016). Only one of the five purified EPS (E142) significantly increased expression of activation markers on BM-DCs. The purified EPS elicited minimal cytokine production in BM-DCs, with the exception of EPS E142. The reason for the immune responsiveness of E142 EPS is not known but might involve recognition by CLRs as mentioned above. Importantly, the purified EPS was checked for lipopolysaccharide (LPS) contamination using the limulus amoebocyte assay which was reported to be below 0.1 EU (endotoxin units, ~0.01 ng) per μg of EPS. Contamination of EPS with LPS is an important potential confounding factor in studies of immunomodulation by EPS due to its strong capacity to activate the NF-κβ pathway through TLR4 signaling.

Polysaccharide capsules associated with pathogenic bacteria are known to sterically shield antigens form opsonizing antibodies, protect against antimicrobial peptides and modulate immune responses (Moxon and Kroll, 1990; Meijerink et al., 2012). Similar, effects can be expected of cell surface associated EPS from lactobacilli and bifidobacterial as shown for the EPS of *Lactobacillus rhamnosus* GG (Lebeer et al., 2010). EPS of commensal bacteria other than lactobacilli and bifidobacteria have been shown to have strong immunomodulatory effects in the gut *in vivo*. Non-toxin producing *Bac. fragilis* strains producing PSA influence the spatial and temporal development of T cell responses during colonization of germ-free mice. This is due to the zwitterionic nature of PSA which is degraded in the phagolysosome by oxidative depolymerisation to yield fragments that can be presented by the MHC class II molecule to CD4+ T cells carrying αβ-T cell receptors. PSA can protect against colitis by a mechanism involving the induction of regulatory T cells and IL-10 production (Mazmanian et al., 2005). Another example of an anti-inflammatory EPS is that produced by the HTF-F strain of *F. prausnitzii* (Rossi et al., 2015). The purified EPM had anti-inflammatory effects on the clinical parameters measured in the mouse DSS model. The purified EPS itself did not induce cytokines or activate human DCs, it reduced the secretion of pro-inflammatory IL-12p70 and increased the production of IL-10 by DCs stimulated with *L. plantarum* (Rossi et al., 2015).

### Role of EPS in bacterial adhesion

The adhesion to the intestinal epithelium, mostly at the colonic level, is thought to help probiotic bacteria to transiently colonize and persist during longer periods in the gut which, in turn, will difficult the colonization of the intestinal pathogens. Therefore, the adhesion to intestinal epithelial cells or to the mucus layer covering this epithelium is one of the properties frequently tested to search for potential probiotic strains (FAO-WHO, 2006). The surface-associated EPS of specific strains of *Lactobacillus* and *Bifidobacterium* might help maintain viability of bacterium during gastrointestinal transit by serving as a protective layer against harsh conditions, such as low pH in the stomach and bile salts in combination with pancreatic enzymes in the duodenum. Once the EPS-producing bacteria reach the colon, these surface macromolecules are involved in the interaction with the intestinal mucosa, with both positive and negative effects on the adhesion reported in the literature (Castro-Bravo et al., 2018).

The mechanism by which the EPS are involved in bacterial adhesion has not been conclusively demonstrated so far. Several methods to *in vitro* quantify the adhesion of EPS-producing bacteria to the intestinal epithelium are used, such as: (1) the metabolic labeling of the bacterium using ^3^H nucleotides (Ruas-Madiedo et al., 2006), (2) the bacterial labeling with fluorescence probes (Živković et al., 2015), (3) the expression of fluorescent proteins in the bacterial surface (Castro-Bravo et al., 2017), and (4) counting the number of bacteria adhered by conventional culture dependent techniques. Animal models have been used to analyse the colonization and persistence of EPS-producing lactobacilli and bifidobacterial (discussed below), but none have verified the production of the polymers *in situ*. However, the zwitterionic PSA produced by *Bac. fragilis* was shown to be produced *in vivo* in mice, by incorporating small functional azide group into the polymer (i.e., GalNAz instead of the GalNAc) using the endogenous biosynthetic machinery of the bacterium. The azide group permitted the incorporation of a fluorescent label, which could then be used to detected and quantify the fluorescent bacteria *in vivo* (Geva-Zatorsky et al., 2015).

In general, the presence of EPS surrounding the bacterial surface, mainly those having a high molar mass, might reduce adherence to intestinal cells, and even to abiotic surfaces, due to shielding of surface macromolecules acting as adhesins. For example, deletion of the cps2-like gene cluster on *L. plantarum* Lp90 improved its adhesion to Caco2 cells in comparison with that of the parental strain (Lee et al., 2016). Lactobacillus johnsonii strain FI9785, which produces two different polymers (EPS-1 and EPS-2), has reduced capacity to form biofilms on different abiotic surfaces compared to isogenic mutant strain (ΔepsE), which does not produce EPS-2. Moreover, a spontaneous “smooth” mutant (epsCD88N), which has an increased accumulation of both polymers, adhered less than the wild-type strain (Dertli et al., 2015). Furthermore, adhesion studies on chicken gut explants using the same strains gave essentially the same results supporting the hypothesis that EPS may hinder molecular interactions with host epithelial membranes (Dertli et al., 2015). Another study with a pair wild-type (NCC533)/isogenic-mutant strains of *L. johnsonii* showed that the non-EPS producing mutant persisted longer in mice following oral administration (Denou et al., 2008). A similar approach was followed for the *B. longum* 105-A strain, where a knockout mutant of a putative priming-GTF gene (cpsD), was constructed. TEM images showed the wild-type strain was surrounded by a dense capsular layer, which was absent in the ΔcpsD mutant, revealing the presence of multiple fimbriae (Tahoun et al., 2017). The wild-type adhered less to Caco-2 epithelial cells than the mutant strain, leading the authors to conclude that the presence of capsular EPS negatively correlates with the presence of adherent factors (Tahoun et al., 2017). In parallel we reached a similar conclusion, but also showing that the molecular weight of the polymer is a key factor influencing the cellular adherence capacity of different EPS-producing strains. Our working model was *Bifidobacterium animalis* subsp. lactis type strain (DSM10140) in which we constructed an isogenic mutant strain (S89L) which differs to wild-type in a single nucleotide in the gene involved in the chain-length determination. The mutant S89L has a “ropy” phenotype due to the higher production of a high molecular weight (HMW), rhamnose-rich EPS fraction (about 103 kDa, 50% L-rhmanose in the hexasaccharide repeating unit) in comparison with the parental strain (Castro-Bravo et al., 2017). Both strains were genetically modified to express mCherry and used to study the adhesion to the human intestinal cell line HT29 and to evaluate their capacity to form a biofilm on different abiotic surfaces. Several qualitative and quantitative techniques showed that the ropy S89L mutant strain had reduced cellular adhesion and attenuated biofilm formation on gold, glass or polystyrene surfaces than the parenteral strain. Taken together these results demonstrate that the presence of the HMW-EPS reduces the adhesion capacity of the strain *in vitro*, which may also have negative impact on intestinal colonization (Castro-Bravo et al., 2017). In fact, the same conclusion was previously reported for L. rhamosus GG (using Caco-2, mucin, and polystyrene) through deletion of the priming-GTF, which abolished the synthesis of a long, galactose-rich EPS (Lebeer et al., 2009). Although *in vitro* the non-EPS producing mutant has good adhesion ability, an *in vivo* study showed that it was more sensitive toward host innate defenses; thus the EPS covering the parental strain GG favors its persistence in the murine gut (Lebeer et al., 2010). Similarly, the EPS covering *B. breve* UCC2003 helps to tolerate the gastrointestinal transit and promote *in vivo* persistence, but not colonization, because the strain is able to evade B-cell responses (Fanning et al., 2012). Finally, it was shown that the Δ*cpsD* mutant of the strain *B. longum* 105-A was internalized by the murine macrophage cell line RAW 264.7, whereas the presence of the EPS prevents efficient phagocytosis (Tahoun et al., 2017). Similar roles of polysaccharide capsules in avoidance of phagocytosis, innate immunity and immunomodulation are well described for Gram-positive opportunist pathogens (e.g., Meijerink et al., 2012).

Therefore, the coverage of the surface of lactobacilli and bifidobacteria by EPS with specific physical-chemical characteristics, e.g., long chain polymers, plays a pivotal role in the interaction of these bacteria with the host.

## EPS interactions with the intestinal microbiota

### Intestinal microbiota

The microbiota is the collective term for the microorganisms present in a defined environment (Marchesi and Ravel, 2015). The human intestinal ecosystem is one of the most densely populated and highly diverse microbial environment known to date, composed of stable and variable microbial groups. The colon contains the highest microbial load in our body (about 10^11^-10^12^ microbial cells/g of intestinal content). Bacteria, archaea, fungi, protozoa and viruses are the main inhabitants of our intestine, in which they stablish commensal, mutualistic or parasitic relationships with the host that shape our physiology. Among them, bacteria are the most abundant members, being *Firmicutes* and *Bacteroidetes* the main bacterial phyla, although other less abundant, such as *Actinobacteria* or *Proteobacteria*, are also present. All together, these microbes provide us with a significant metabolic potential encoded in the metagenome; thus the intestinal microbiota has been postulated as another organ of the human body (Sánchez et al., 2017). More than a decade has passed since the first works stablishing links between microbiota profiles and different diseases, and today we know that more than 100 diseases and disorders have been associated with changes in the microbiotas of the human body (Rojo et al., 2017). This relevant milestone in microbiome science has been made possible thanks to the advent and refinement of Next-Generation Sequencing (NGS) methodologies, which allow the genomic analysis of microbial samples without the need of cultivation. In this way, we know that aberrant microbiota profiles have been found in highly prevalent diseases, such as inflammatory bowel diseases (IBD), irritable bowel syndrome (IBS) or colorectal cancer, among others (Gevers et al., 2014; Bullman et al., 2017; Bennet et al., 2018).

In general, the presence of *Lactobacillus* and *Bifidobacterium* within the intestinal microbiota has been linked to a healthy state of the host. These two genera do not share the same niche; while *Lactobacillus* is a common member of the small intestine of humans and other mammals (Stearns et al., 2011; Xiao et al., 2015), *Bifidobacterium* represents one of the most abundant genera in the human colon, especially in infants (Arumugam et al., 2011; Milani et al., 2017). Low levels of these two bacterial groups have been correlated with specific health problems. For instance, reduced levels of bifidobacteria and lactobacilli are found in IBS (Liu et al., 2017), and a decreased abundance of bifidobacteria, in particular the *Bifidobacterium bifidum* species, is detected in the microbiota of ulcerative colitis patients (Duranti et al., 2016). On the contrary, the addition of bifidobacteria and/or lactobacilli as a probiotic supplement can help to balance an aberrant intestinal microbiota (Staudacher et al., 2017). Thus, it seems that these two microorganisms could play a role in maintaining the normal functions of our intestinal ecosystems.

There is fragmentary information about the interaction of EPS from lactobacilli or bifidobacteria with other gut microbial members. Among these interactions, the antagonistic effect against bacterial pathogens and the capacity of EPS to be used as substrates for other enteric bacteria, are two activities that have been studied in some detail.

### EPS as nutrient source

EPS synthesized by LAB and bifidobacteria could have a potential role as prebiotics (Salazar et al., 2016) whose consensus definition was recently accepted as “substrate that is selectively utilized by host microorganisms conferring a health benefit” (Gibson et al., 2017). For orally delivered prebiotics, a requirement for a substrate to be considered as such is that it should not be digested nor absorbed in the upper part of the gastrointestinal tract and arrive to the colon where it can be utilized by specific (beneficial) members of the gut microbiota (Roberfroid et al., 2010).

It is hypothesized that bacterial HoPS could reach the colon almost undigested thanks to their chemical composition, similar to inulin and FOS (fructooligosaccharide) prebiotics. In this case their large molecular weight could difficult the enzymatic hydrolysis, although the lack of human enzymatic activities specific for these polymers could also be a plausible explanation. Additionally, several studies have demonstrated that the simple primary structure allows them to be fermented by fecal microbiota. As an example, is the case of the HoPS isolated from *Lactobacillus sanfranciscensis* which had a bifidogenic effect being able to modify the gut microbiota composition (Dal Bello et al., 2001). The metabolism by bifidobacteria of HoPS produced by *L. sanfranciscensis* during single species dough fermentation was also studied in order to evaluate the prebiotic properties of fermented foods. For that, the water soluble polysaccharide fraction was extracted from the fermented sourdough and used to grow different bifidobacteria. The results showed that all bifidobacteria tested were able to metabolize the β-fructan synthesized by this lactobacilli (Korakli et al., 2002; Galle and Arendt, 2014). These results seem to indicate that HoPS could be used as fermentable substrates by the intestinal microbiota due to their simple primary structure; however, the evidence for this is lacking from *in vivo* studies.

In the case of HePS, their more complex structure could lead to a high resistance to gastrointestinal digestion but this has not been demonstrated *in vivo*. Wistar rats fed with EPS-producing *B. animalis* subsp. *lactis* or *B. longum* showed an increase in the levels of intestinal bifidobacteria analyzed in caecum content and feces (Salazar et al., 2011) suggesting that their polymers could arrive intact to the colon. Similarly, the persistence of the EPS-producing *L. rhmanosus* GG in the gut was higher, than its mutant strain lacking the polymer, due to the protective role of the HePS against immune innate factors (Lebeer et al., 2010). *In vitro* models also showed that the susceptibility of HePS to biodegradation in the gut depends on the physical-chemical composition of the polymer. In general, simplest structures of the repeating units building these polymers are easily hydrolysed by enzymes. As example, the HePS from *Streptococcus thermophilus* Sfi39 and Sfi12 were degraded in higher extent than those of *Lactobacillus sakei* 0-1 and *Lactobacillus helveticus* Lh59, which had more complex structures (Ruijssenaars et al., 2000). Additionally, HePS purified from different bifidobacterial species from human origin modified the composition of the microbiota when added to *in vitro* fermentations (Salazar et al., 2008, 2009).

In summary, HoPS and HePS synthesized by *Lactobacillus* and *Bifidobacterium* seem to be resistant to gastrointestinal digestion, but they are susceptible of biodegradation by the intestinal microbiota, this feature being dependent on the physicochemical characteristics of the EPS as well as on the pool of glycolytic enzymes harbored by gut microorganisms. Besides, monosaccharides released from biodegradable EPS could also have influence on microbiota composition through cross-feeding mechanisms (Rios-Covián et al., 2016). Thus, HoPS and HePS could be considered as prebiotic substrates acting as carbon source for colon inhabitants, where starving conditions limit growth, rendering to a healthy balanced microbiota. Therefore, the host can benefit from microbial EPS metabolism (Salazar et al., 2016).

### EPS as microbial antagonists

The antagonistic effect of EPS-producing lactobacilli and bifidobacteria, and in particular cases their isolated EPS, against microbial pathogens has been studied using *in vitro* and *in vivo* models. One of the traditional phenotypic features tested during *in vitro* probiotic characterization is the ability to co-aggregate with pathogens, since it is believed that co-aggregation decreases the accessibility of the harmful microorganisms to the intestinal epithelium. In this regard, high EPS producing strains of *Lactobacillus delbrueckii* subsp. *bulgaricus* were shown to be associated with a high co-aggregation ability with *Escherichia coli* (Aslim et al., 2007). More complex models, involving cell lines, have also been used to shed light on the antagonistic effects of EPS producing strains. Some examples include the attenuation of enterotoxigenic *E. coli-*induced inflammatory response by the EPS of *L. delbrueckii* TUA4408L on porcine intestinal enterocytes (Wachi et al., 2014), or the capability of the EPS produced by lactobacilli and bifidobacteria from human and dairy origin to decrease the cytotoxic effect of *Bacillus cereus* toxins on the monolayer integrity of Caco-2 human colonocytes (Ruas-Madiedo et al., 2010). Particularly interesting are the experiments carried out with isogenic mutants, in which the activity of EPS producing and non-producing strains, or strains producing different types or EPS, are compared in the same genetic background. The activity against *Candida* of *L. rhamnosus* GG, its EPS deficient mutant, and the purified EPS was analyzed showing that the EPS layer might be involved in the reduction of hyphal formation and in the competition with *Candida* during adhesion to vaginal epithelial cells (Allonsius et al., 2017). Also, the protective effect of EPS-producing *Lactobacillus paraplantarum* BGCG11, and its non-EPS-producing isogenic strain NB1, against seven opportunistic pathogens was determined on HT29-MTX cell monolayers, pointing out that the EPS of the parental BGCG11 strain could be involved in the reinforcement of the innate mucosal barrier by hindering the contact of pathogens, such as *E. coli* or *Listeria monocytogenes*, with the epithelial cells (Živković et al., 2016). Another study with a *Lactobacillus paracasei* subsp. *paracasei* BGSJ2-8 EPS-deficient mutant, producing an EPS with a different composition than the wild type, indicated that the wild type EPS is essential to reduce *E. coli* association with Caco-2 cells, while the mutant strain was not able to counteract this interaction (Živković et al., 2016). This highlights the importance of EPS composition, and not only EPS production, in the potential interaction with pathogens.

Regarding *in vivo* experiments, different animal models have been used to study how EPS-producing strains interfere with pathogen activity. For instance, *L. johnsonii* FI9785, a poultry-derived isolate producing two different exopolysaccharides, was effective to supress colonization and persistence of *Clostridium perfringens* in poultry and to reduce colonization by *E. coli* of the small intestine (La Ragione et al., 2004; Dertli et al., 2013). Also, *B. longum* subsp. *infantis* 35624, which synthesizes an EPS containing galactose, glucose, galacturonic acid, and 6-deoxy-L-talose, decreases the effect of *Salmonella* on brush border enzyme activity and weight loss in mice (Symonds et al., 2012; Altmann et al., 2016). Although these studies show a correlation between the administration of EPS-producing strains and a reduction in the deleterious activity of some pathogens, the precise role of the EPS, and its causality, cannot be unequivocally demonstrated. In this regard, the results obtained with *B. breve* UCC2003, and their isogenic non-EPS producing isogenic mutant, are those providing more solid evidence of the role of EPS in pathogen antagonism (Fanning et al., 2012). These authors demonstrated that the surface-attached EPS of strain UCC2003 aids in long-term persistence of the bacteria in the intestine of mice and provides the host with protection against the enteric murine pathogen *C. rodentium*, suggesting a role of EPS in conferring the health benefits normally associated with probiotic strains.

## Concluding remarks (Box 1)

EPS produced by *Lactobacillus* and *Bifidobacterium* might act as effector surface macromolecules that are directly involved in the interaction of these bacteria with different host cells as well as with the microbial community that occupy the intestinal lumen. In order to understand these complex interactions is pivotal to gain more insight into the biosynthetic machinery of these polymers, as well as to determine which are the key properties of the EPS that are important for their biological functions. Thus, combination of high-throughput techniques allowing to take advantage of the information codified in the genomes of the producer strains, in combination with powerful analytical techniques and *in vitro* models better reflecting the *in vivo* physiology of the host, will be valuable tools for the establishment of this correlation genes-structure-function. This knowledge would facilitate use of synthetic biology to obtain tailor-made polymers, opening up possibilities to enhance attributes considered positive for health. It would allow to improve the production of polymers with known benefits given that, due to their low production yield, the application of purified polymers is not affordable to date. Furthermore, the biodiversity of EPS synthesized by bacteria from nature can be further explored to find novel biotechnological applications. Thus, research on bacterial EPS is a relevant topic with considerable prospects for health and biotechnological applications.

Box 1 **Concluding remarks.**

***Well-known statements***

- The genetic determinants for EPS synthesis have been identified
- The physical-chemical properties of EPS determine their biological properties
- EPS help the survival of the producing bacteria under the gastrointestinal challenges
- The EPS surrounding the bacterial surface reduces adherence to intestinal cells due to shielding of surface macromolecules acting as adhesins
- Isogenic EPS-producing and non-EPS-producing strains induce different immune responses
- EPS are resistant to gastrointestinal digestion and they are susceptible of biodegradation by the intestinal microbiota
- Specific EPS-producing strains act as antagonists of pathogens in the gut

***Future perspectives***

- To gain insight in the biosynthetic route of EPS formation in *Lactobacillus* and *Bifidobacterium*
- To know whether, or not, bacterial EPS activate or inhibit innate immunity via specific pattern-recognition receptors
- To decipher the mechanisms behind the beneficial properties of EPS
- To *in vivo* demonstrate the EPS production by intestinal lactobacilli and bifidobacteria.

## Author contributions

JMW, AM, and PR-M contributed with the organization and structure of the review and NC-B, JMW, AM, and PR-M contributed to the writing. PR-M was in charge of organizing the drafted manuscript and all authors performed a critical revision of the manuscript and approved the final version.

### Conflict of interest statement

The authors declare that the research was conducted in the absence of any commercial or financial relationships that could be construed as a potential conflict of interest.
